# Distal Triceps Tendon Ruptures: A Case Series Highlighting Diagnostic Challenges, Surgical Management, and Functional Outcomes

**DOI:** 10.7759/cureus.81990

**Published:** 2025-04-10

**Authors:** David H Wong, Neil Ashwood

**Affiliations:** 1 Trauma and Orthopaedics, University of Leicester, Leicester, GBR; 2 Trauma and Orthopaedics, University Hospitals of Derby and Burton, Derby, GBR

**Keywords:** elbow injuries, flake sign, suture anchor repair, triceps tendon rupture, ultrasonography in tendon injuries

## Abstract

Triceps tendon ruptures are uncommon but significant injuries, often presenting diagnostic challenges due to nonspecific clinical features. Delayed or missed diagnoses can result in prolonged functional impairment. We present a retrospective case series of five patients who sustained distal triceps tendon ruptures through various mechanisms, including direct trauma and heavy lifting. Clinical presentations ranged from elbow swelling and pain to palpable tendon gaps, with radiographic "flake signs" consistently observed. Ultrasonography effectively identified tendon ruptures and retraction in four cases, reinforcing its diagnostic value. All patients underwent surgical repair using suture anchor techniques, achieving satisfactory outcomes. This case series emphasises the importance of early recognition, timely imaging, and prompt surgical intervention in managing distal triceps tendon ruptures to optimise patient recovery.

## Introduction

Triceps tendon ruptures are uncommon, representing only 0.8% of all tendon injuries, thus being among the rarest tendon injuries encountered clinically [1]. Due to their rarity, triceps tendon ruptures are frequently missed due to a low degree of suspicion from clinicians. When combined with nonspecific signs such as posterior elbow pain and swelling, this often leads to delayed or incorrect diagnoses [2]. The triceps brachii muscle, consisting of long, lateral, and medial heads, primarily facilitates elbow extension [3]. Ruptures typically occur distally at the tendon’s insertion onto the olecranon [4]. Given that the triceps accounts for 55% of upper limb muscle mass, early and accurate diagnosis is crucial to ensure functional arm movement [5]. Timely diagnosis is critical, as it allows for prompt surgical repair within three weeks of injury, where primary repair is always viable. Beyond this timeframe, reconstruction using autografts or allografts may be necessary due to tendon retraction and scarring, resulting in a more complex procedure with a prolonged postoperative recovery [6,7]. Furthermore, delayed surgical intervention can lead to chronic impairment of elbow function, such as loss of function, decreased strength, muscle atrophy and chronic pain [8-11]. Therefore, promptly recognising injury patterns and clinical manifestations is critical. In this case series, we discuss five cases of distal triceps tendon ruptures resulting from direct trauma, focusing on the diagnostic challenges, surgical interventions, and patient outcomes.

## Case presentation

Case 1

**Figure 1:**
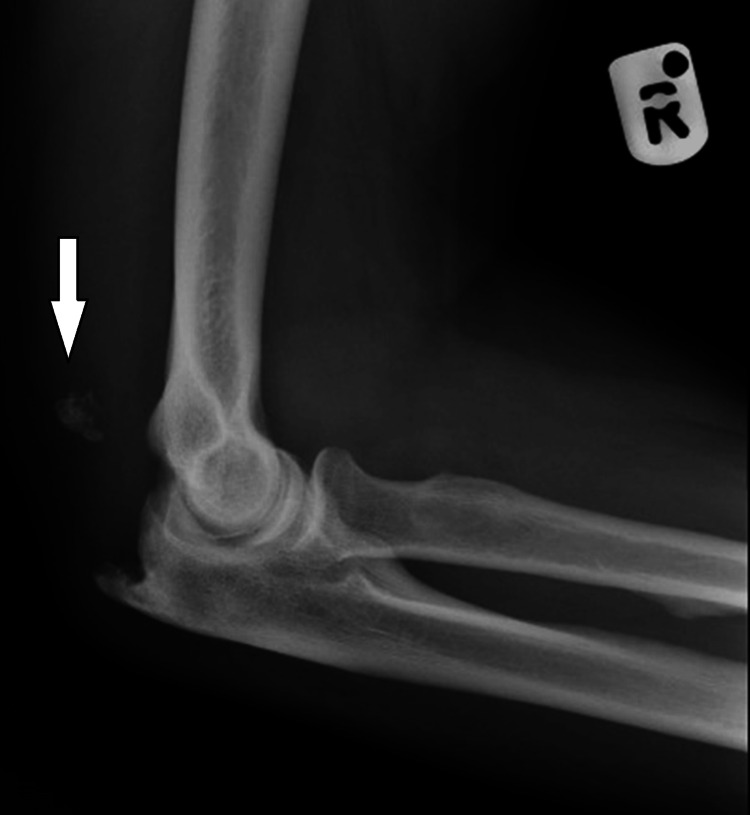
Lateral elbow radiograph showing calcific fragments (white arrow) posterior to the distal humerus, indicative of avulsion fractures from the olecranon.

A 58-year-old male presented to the emergency department four days after falling onto his right elbow, experiencing pain and bruising. Clinical examination revealed olecranon tenderness, swelling, and painful active elbow extension, but no palpable tendon gap. Neurovascular status was intact. Initial radiographs identified calcific fragments near the distal humerus (Figure 1). The patient was managed conservatively with a collar-and-cuff sling and referred to the fracture clinic.

**Figure 2:**
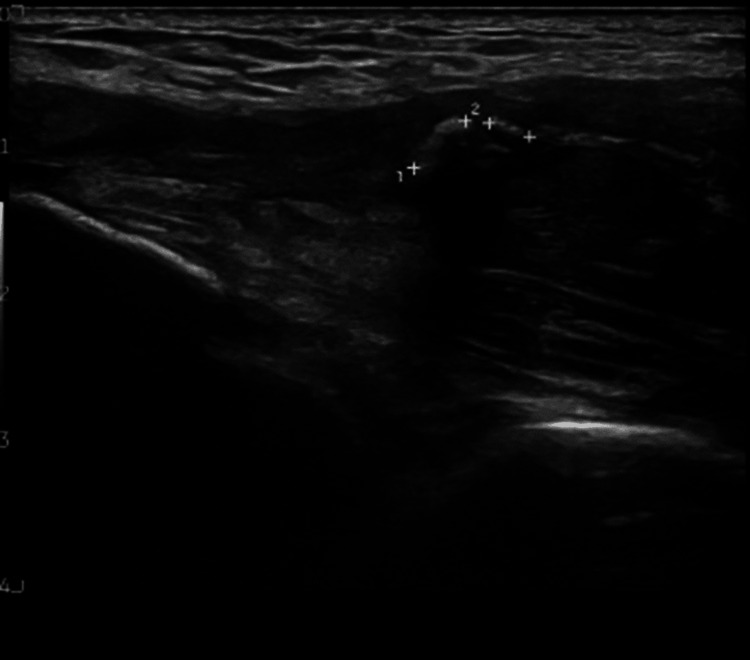
Ultrasound image of the right elbow showing a near full-thickness distal triceps tendon tear, with notable proximal retraction (marked by calipers).

Nine days later, ultrasonography confirmed a nearly complete tear of the distal triceps tendon at the tendo-osseous junction, with proximal tendon retraction of 31 mm. Additionally, the tendon was thickened, exhibiting six echogenic calcifications ranging from 2.5 to 3.7 mm (Figure 2). Surgical repair was performed seven weeks post-injury, utilising two JuggerKnot 2.9 mm anchors (Zimmer Biomet, Warsaw, IN, USA). Postoperatively, the elbow was immobilised in a backslab for two weeks, followed by progressive extension using a hinged elbow brace for four weeks. Disabilities of the Arm, Shoulder and Hand score (DASH score) was reported as 26 post-operatively, highlighting only mild to moderate functional disability.

**Figure 3:**
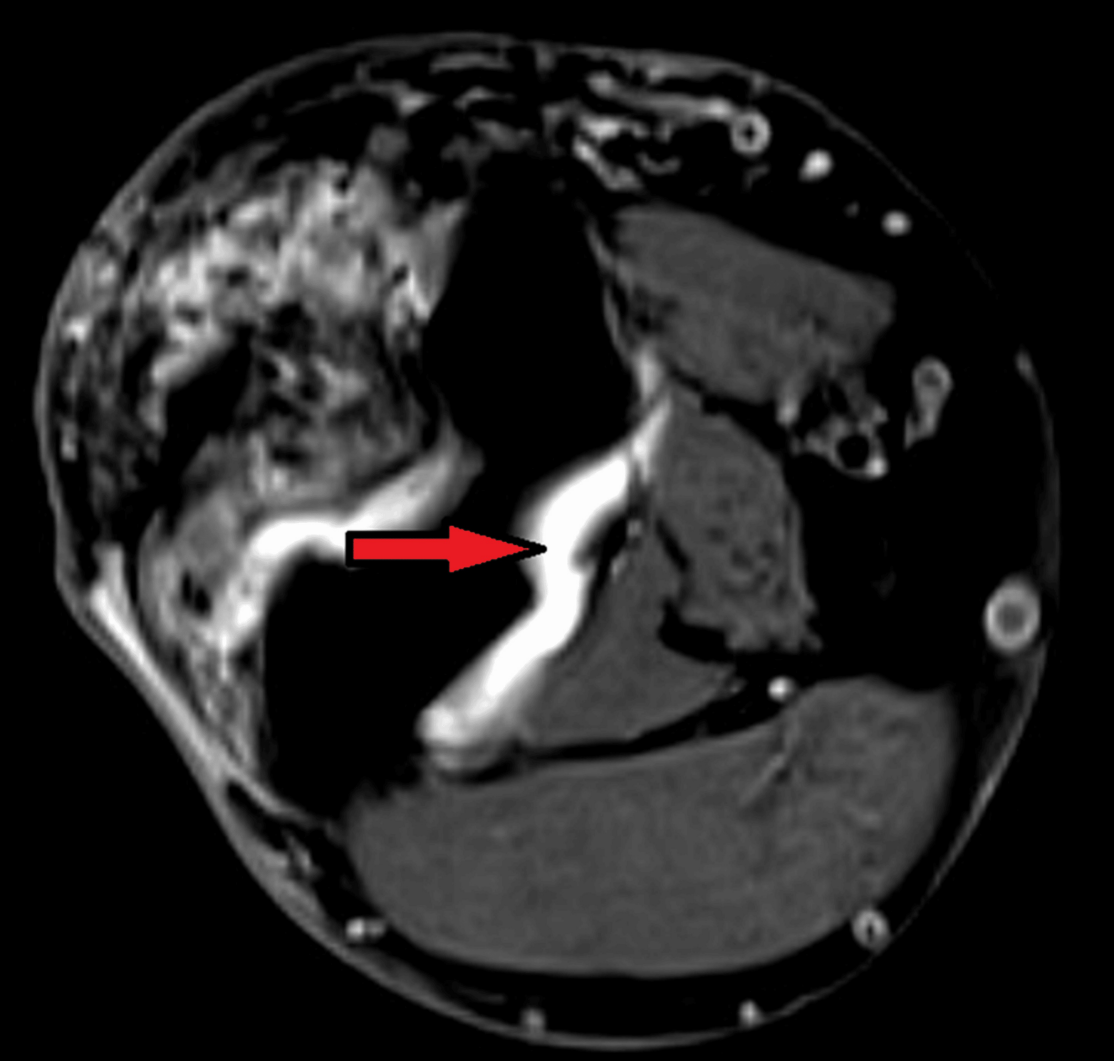
The axial MRI of the right elbow (the red arrow) indicates a hyperintense fluid signal between the triceps tendon and the olecranon insertion, suggestive of a partial re-tear or incomplete tendon healing.

Six months post-surgery, following another fall onto the same elbow, MRI raised concerns for a potential re-tear due to a 4 mm fluid plane at the medial portion of the tendon insertion; however, the surgical repair remained intact, and additional surgery was not required (Figure 3). Despite one year of physiotherapy, the patient continued to experience functional limitations, with elbow flexion restricted to 95 degrees and extension limited to 30 degrees. Consequently, he was scheduled for a right arthroscopic elbow procedure. At two years post-injury, spontaneous improvement occurred, resulting in normalised elbow stiffness and range of motion, and further surgical intervention was no longer required.

Case 2

**Figure 4:**
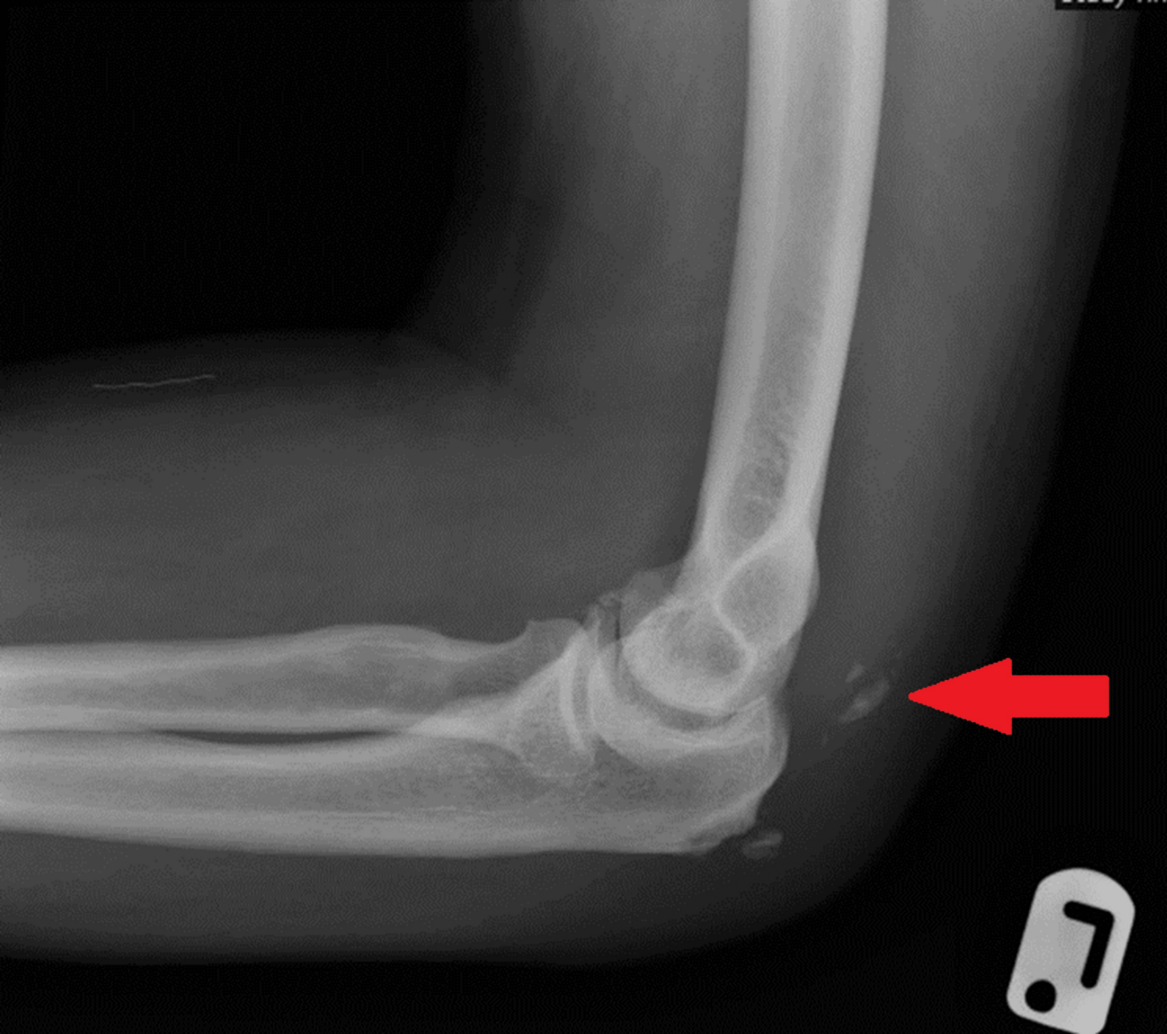
Lateral radiograph of the left elbow demonstrating a comminuted olecranon fracture with notable diastasis. The red arrow highlights the retracted fracture fragments.

A 68-year-old male presented with significant swelling extending from the left elbow to the hand, six days after a fall onto the elbow. Clinical examination revealed a warm, swollen elbow with localised tenderness and notably reduced active extension. Neurovascular status remained intact. The patient had a medical history notable for gout. Initial radiography demonstrated a comminuted olecranon fracture with clear diastasis and fragment retraction (Figure 4).

**Figure 5:**
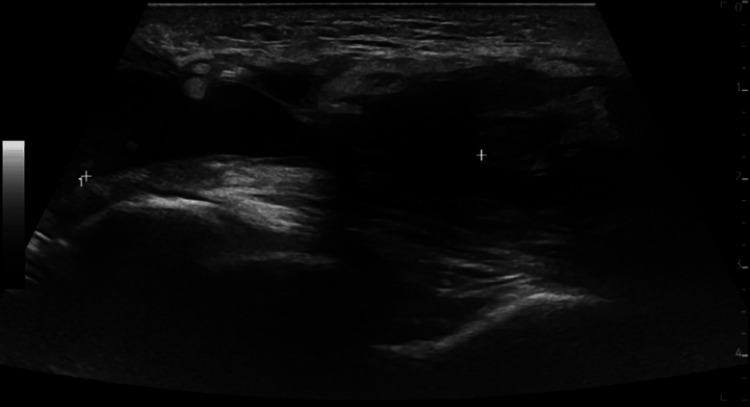
Ultrasound image of the left elbow demonstrating complete avulsion of the posterior triceps tendon component, with significant proximal retraction (5 cm) marked by calipers.

Subsequent ultrasonography confirmed a complete avulsion of the posterior component of the distal triceps tendon, accompanied by proximal retraction measuring approximately 50 mm (Figure 5). The patient underwent open surgical repair using suture anchors combined with cubital tunnel release within three weeks post-injury. DASH score was reported as 18 post-operatively, highlighting mild functional disability. Postoperative management involved elbow immobilisation in a cast for one week, transitioning to a splint for an additional four weeks. Physiotherapy was not required. The recovery was uneventful, without complications or evidence of re-rupture, and the patient was discharged at three months with restored elbow function.

Case 3

**Figure 6:**
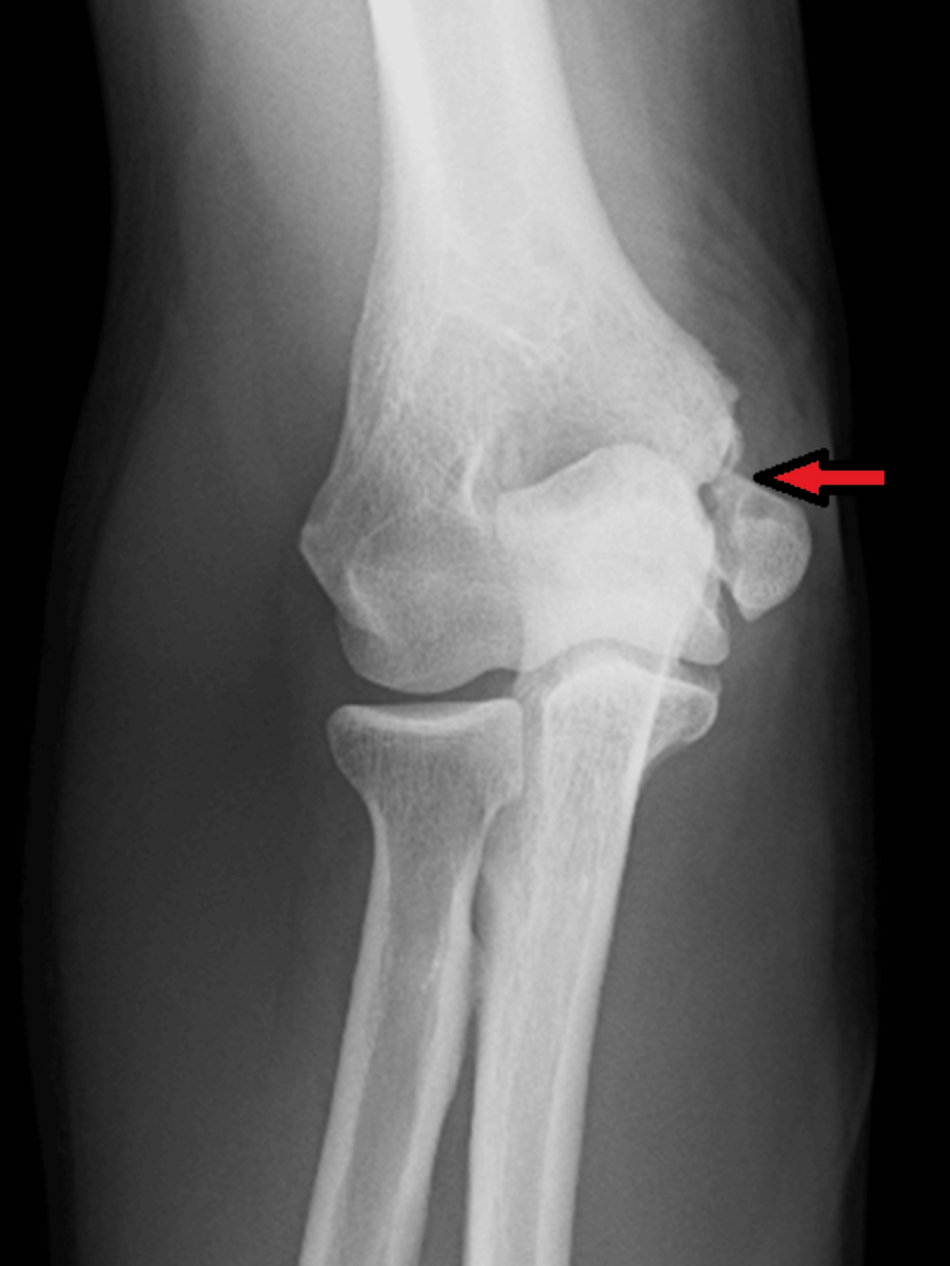
Anteroposterior (AP) view of right elbow from showing several tiny calcific densities are noted lying adjacent to the posterior aspect of the tip of the olecranon process of the proximal ulna (red arrow).

A 54-year-old male presented with acute pain and swelling around the right elbow after experiencing a sudden "pop" sensation while pulling a washing machine. Clinical examination revealed tenderness over the olecranon, significant swelling, and painful active elbow extension, although no palpable tendon gap was detected. The neurovascular examination was unremarkable. Radiography identified avulsion fragments posterior to the olecranon, consistent with an avulsion fracture (Figure 6). Ultrasonography confirmed a full-thickness triceps tendon tear at the tendo-osseous junction, with 24 mm of proximal retraction.

The patient underwent surgical repair within five days post-injury, utilising a posterior surgical approach and fixation with two SwiveLock anchors (Arthrex Inc., Naples, FL, USA). Postoperatively, the elbow was immobilised in a polysling. Five days after surgery, the patient returned to the emergency department following another fall that resulted in renewed elbow pain and swelling; however, ultrasonography ruled out a re-tear. DASH score was reported as 21 post-operatively, highlighting only mild functional disability. However, recovery proceeded without complications, and at the three-month follow-up, the patient had regained full elbow strength and range of motion and was subsequently discharged.

Case 4

**Figure 7:**
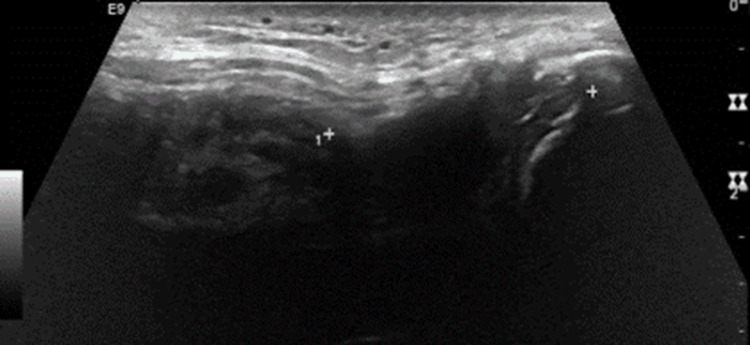
Ultrasound image of the left elbow demonstrating a full-thickness triceps tendon tear, with a tendon deficit measuring 2.8 cm from the olecranon. A tendon stump is visible at the insertion (marked by calipers).

**Figure 8:**
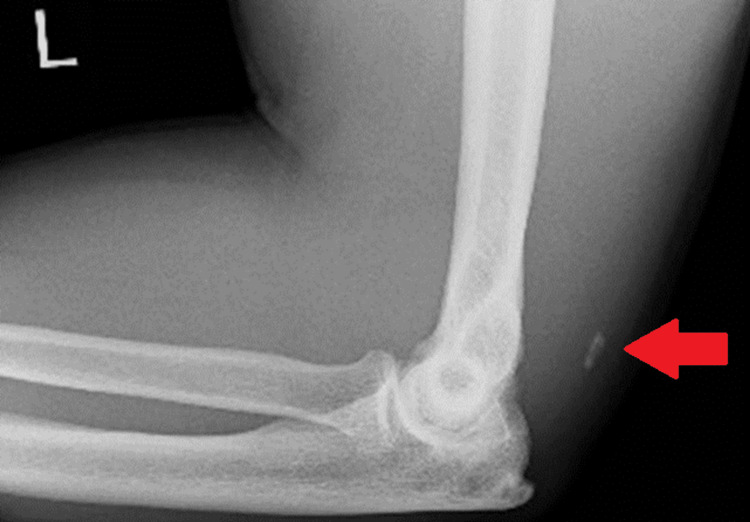
Lateral radiograph of the left elbow demonstrating a prominent olecranon spur and calcification posterior to the distal humerus, consistent with a "flake sign" (red arrow).

A 52-year-old male presented following a slip-and-fall injury onto his left arm. Clinical examination revealed tenderness over the olecranon and distal humerus, painful active elbow extension, and associated shoulder discomfort. Radiographic evaluation of the shoulder was normal. However, an ultrasound examination performed three days post-injury identified a full-thickness musculotendinous tear of the left triceps, with notable calcification at the insertion site on the olecranon (Figure 7). Additionally, radiography demonstrated a small calcific fragment anterior to the olecranon (Figure 8).

Open surgical repair of the triceps tendon was performed one week following the injury, using two GII suture anchors (DePuy Mitek, Raynham, MA, USA). Postoperative immobilisation and rehabilitation protocol were consistent with the approach described in Case 1. At the six-month follow-up, ultrasonography revealed persistent calcific tendinopathy, but MRI ruled out a re-tear. One year postoperatively, the patient exhibited an excellent functional outcome with a DASH score of 8, with full elbow range of motion and triceps strength equal to the uninjured side.

Case 5

**Figure 9:**
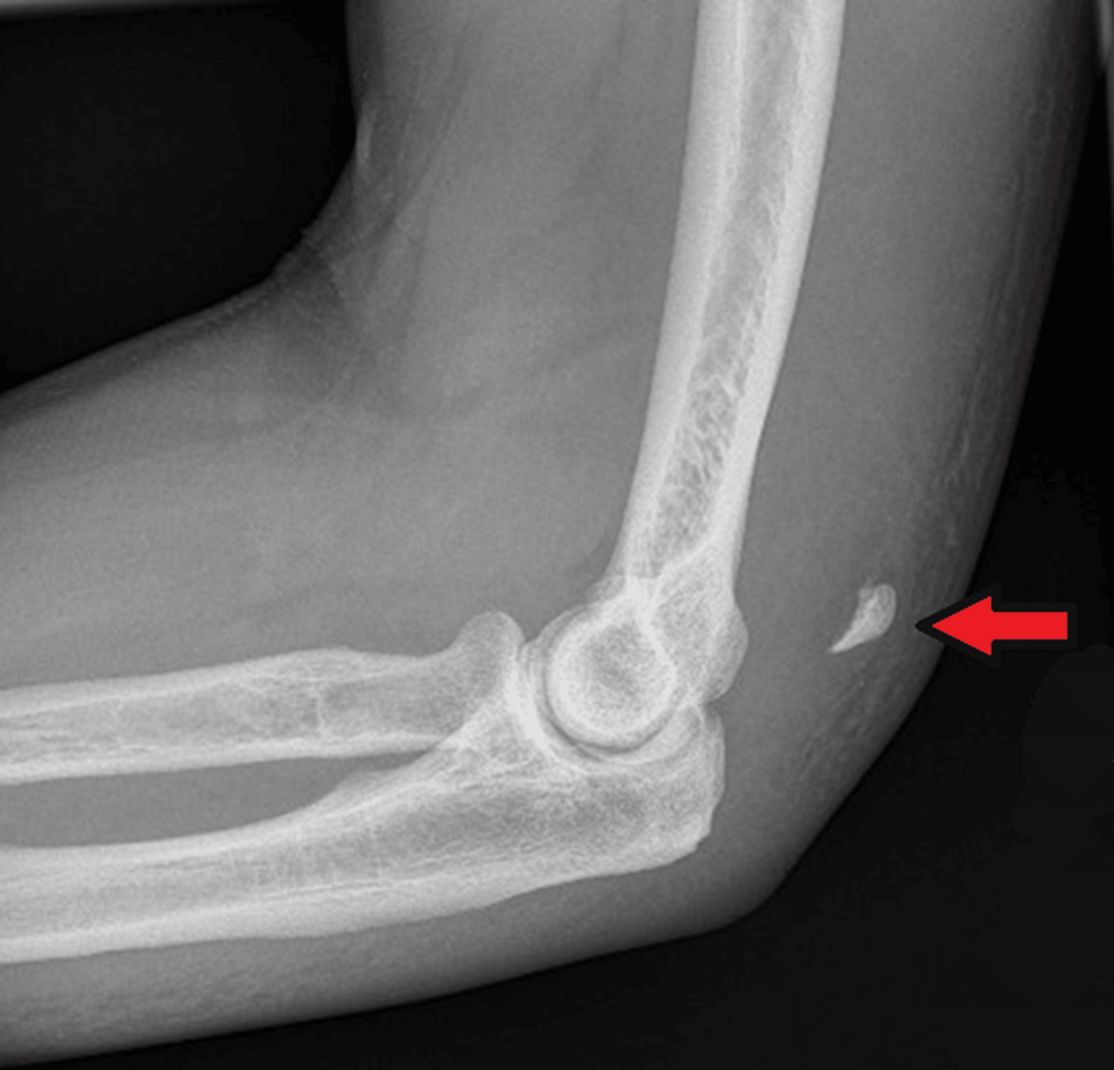
Lateral radiograph of the left elbow demonstrating a small avulsed bony fragment adjacent to the posterior distal humerus, consistent with a proximally retracted olecranon avulsion fracture (red arrow).

A 66-year-old male presented after tripping and falling onto his left elbow. Clinical examination revealed elbow swelling, painful active elbow extension, and a palpable gap at the tendon insertion. He had a notable medical history of rheumatoid arthritis. Initial radiography identified a small bony fragment posterior to the distal humerus, consistent with an avulsion fracture of the olecranon (Figure 9). Ultrasonography was not performed.

Surgical repair was conducted 10 days after injury using suture anchor fixation. Postoperatively, the elbow immobilisation and rehabilitation protocol mirrored that of Case 1. DASH score was reported as 22 post-operatively, highlighting mild functional disability. However, following two months of targeted physiotherapy, the patient regained excellent functional outcomes, with forearm extension exceeding 100 degrees. The postoperative course remained uncomplicated, resulting in full recovery of strength and elbow mobility.

We present five cases of distal triceps tendon rupture in male patients aged 52 to 68 years. All cases resulted from direct trauma or forceful exertion and presented with elbow pain, swelling, and reduced extension. Radiographic findings consistently demonstrated the flake sign, while ultrasonography confirmed tendon retraction in four cases. All patients underwent surgical repair using suture anchors, with favourable postoperative outcomes. The results are presented in Table 1.

**Table 1:** Summary of clinical, radiological, and surgical findings in patients with distal triceps tendon rupture. DASH score: Disabilities of the Arm, Shoulder and Hand score

Case	Age	Sex	Mechanism of Injury	Clinical Findings	Imaging Findings	Management	DASH Score Post-operation	Outcomes
1	58-year-old	Male	Fall onto right elbow	Swelling, olecranon tenderness, painful active extension, no palpable gap	Radiography: Calcific fragments, flake sign present; Ultrasound: Full-thickness tear, 31mm retraction, calcifications	Primary surgical repair (suture anchors), immobilisation, physiotherapy	26	Initial functional limitations, full recovery at 2 years without further surgery
2	68-year-old	Male	Fall onto left elbow	Swelling, warmth, tenderness, reduced active extension	Radiography: Olecranon avulsion, flake sign present; Ultrasound: Complete avulsion with 50mm retraction of triceps	Primary surgical repair (suture anchors)with cubital tunnel release, immobilisation	18	Full recovery by 3 months, no complications
3	54-year-old	Male	Pulling a washing machine	Swelling, olecranon tenderness, painful active extension, no palpable gap	Radiography: Avulsion fragments, flake sign present; Ultrasound: Full-thickness tear, 24mm retraction	Primary surgical repair (suture anchors), immobilisation	21	Full strength and range of motion at 3 months
4	52-year-old	Male	Slip and fall onto left arm	Swelling, olecranon tenderness, painful active extension, shoulder pain	Radiography: Small calcific body, flake sign present; Ultrasound: Full-thickness tear, calcifications	Primary surgical repair (suture anchors), immobilisation	8	Excellent range of motion and strength at 1 year
5	66-year-old	Male	Fall onto left elbow	Swelling, painful extension, palpable gap	Radiography: Small bony density, flake sign present; Ultrasound: Not performed	Primary surgical repair (suture anchors), immobilisation, physiotherapy	22	Full recovery of strength and range of motion at 2 months

## Discussion

Triceps tendon ruptures represent uncommon yet clinically significant injuries that pose diagnostic and therapeutic challenges due to their nonspecific presentation and potential for delayed recognition [1,3]. Distal triceps tendon ruptures account for approximately 0.8% of all tendon injuries, making them amongst the rarest tendon injuries encountered clinically [1]. Due to this rarity, clinicians often face diagnostic delays, increasing the likelihood of long-term functional impairment [8-11].

In our case series, triceps tendon ruptures predominantly occurred following direct trauma, consistent with prior studies [12,13]. Clinical signs commonly included swelling, tenderness localised to the olecranon, and painful limitation of active elbow extension [13]. Notably, the classic palpable tendon gap was inconsistently present in our series, likely masked by acute swelling, which contributed to diagnostic challenges [12]. While the modified Thompson squeeze test, as described by Viegas, offers an alternative when the palpable tendon gap is obscured [14], its reliability remains variable. In everyday clinical practice, it has shown to be difficult to perform and still susceptible to being masked by pain and swelling [15].

Although MRI is considered the gold standard for comprehensive triceps tendon evaluation [16], it was employed in only one instance (Case 1) to assess a suspected re-tear post-operatively. In our cohort, initial diagnoses were reliably made using more efficient imaging modalities. Radiographic findings consistently demonstrated the "flake sign," characterised by small avulsed calcific fragments proximal to the olecranon, which is highly indicative of distal triceps tendon avulsions [6,17,18]. More importantly, ultrasonography effectively identified and quantified tendon retraction in 80% of cases (4/5), underscoring its diagnostic value as an adjunctive imaging tool [17,19]. Ultrasonography offers several key benefits over MRI: (a) rapid accessibility, (b) dynamic real-time assessment during patient movement and (c) substantial cost savings. This case series demonstrates that radiography and ultrasonography offer significant practical advantages in the acute diagnostic setting, supporting a tiered imaging approach that maintains diagnostic accuracy while optimising resource allocation.

Literature reveals that transosseous bone tunnels are the most frequently used method in the primary repair of triceps tendon ruptures [18]. However, in our cohort, all patients underwent surgical repair utilising knotless suture anchors in a modified suture-bridging configuration. High-strength sutures were passed in a crisscross manner to optimise load distribution, providing robust fixation while minimising any soft tissue irritation. The average time from injury to surgery was 2.6 weeks, which aligns with established literature that advocates for timely surgical intervention for optimal outcomes [6]. Postoperative management included immobilisation followed by a structured rehabilitation protocol, which facilitated successful recovery, with all patients regaining satisfactory elbow strength and range of motion [7,18]. No significant postoperative complications or re-ruptures occurred, although minor findings such as persistent calcific tendinopathy were observed, which did not affect clinical outcomes. There was an average post-operative DASH score of 19 without re-ruptures, highlighting only residual mild functioning difficulty in our cohort.

This case series reinforces the importance of high clinical suspicion, prompt radiographic evaluation, and targeted ultrasonography in diagnosing distal triceps tendon ruptures. Additionally, our findings support the effectiveness and reliability of suture anchor fixation techniques and structured rehabilitation programs in achieving favourable functional recovery and minimising complications.

## Conclusions

Distal triceps tendon ruptures are uncommon injuries that require a high index of suspicion, particularly after elbow trauma. Early diagnosis, supported by radiographic and ultrasonographic evaluation, is essential for timely intervention. The flake sign on radiographs and ultrasonography findings of tendon retraction are key diagnostic indicators. Surgical repair using suture anchors, followed by structured rehabilitation, provides favourable outcomes, as demonstrated in our series. Overall, prompt recognition and management are crucial to minimising complications and ensuring optimal functional recovery.
